# Emerging Applications of Nanotechnology in Healthcare and Medicine

**DOI:** 10.3390/molecules28186624

**Published:** 2023-09-14

**Authors:** Shiza Malik, Khalid Muhammad, Yasir Waheed

**Affiliations:** 1 Bridging Health Foundation, Rawalpindi 46000, Pakistan; 2 Department of Biology, College of Science, UAE University, Al Ain 15551, United Arab Emirates; 3 Office of Research, Innovation and Commercialization, Shaheed Zulfiqar Ali Bhutto Medical University, Islamabad 44000, Pakistan; 4 Gilbert and Rose-Marie Chagoury School of Medicine, Lebanese American University, Byblos 1401, Lebanon

**Keywords:** nanotechnology, nanobiotechnology, nanomedicine, medical applications, diagnosis, disease treatment, drug-delivery, healthcare

## Abstract

Knowing the beneficial aspects of nanomedicine, scientists are trying to harness the applications of nanotechnology in diagnosis, treatment, and prevention of diseases. There are also potential uses in designing medical tools and processes for the new generation of medical scientists. The main objective for conducting this research review is to gather the widespread aspects of nanomedicine under one heading and to highlight standard research practices in the medical field. Comprehensive research has been conducted to incorporate the latest data related to nanotechnology in medicine and therapeutics derived from acknowledged scientific platforms. Nanotechnology is used to conduct sensitive medical procedures. Nanotechnology is showing successful and beneficial uses in the fields of diagnostics, disease treatment, regenerative medicine, gene therapy, dentistry, oncology, aesthetics industry, drug delivery, and therapeutics. A thorough association of and cooperation between physicians, clinicians, researchers, and technologies will bring forward a future where there is a more calculated, outlined, and technically programed field of nanomedicine. Advances are being made to overcome challenges associated with the application of nanotechnology in the medical field due to the pathophysiological basis of diseases. This review highlights the multipronged aspects of nanomedicine and how nanotechnology is proving beneficial for the health industry. There is a need to minimize the health, environmental, and ethical concerns linked to nanotechnology.

## 1. Introduction

The world is theorized to have accidentally formed via the Big Bang that occurred from an unstable microscopic-sized energized particle (atom). A single bit created an entire universe, and now scientists are working again on similar small particles to create marvels of science. From here, the world of nanoscience has arrived and taken a firm place in every aspect of science and technology [1]. The vision for nanotechnology was presented by Nobel Prize-winning physicist Richard P. Feynman, who proposed the application of more significant objects and mechanistic tools at a smaller tool and particle scale, as he believed that “there is plenty of room at the bottom” [1,2]. Nowadays, apart from physicists, scientists from multiple fields believe that in the future, nanoscale manufacturing technologies and instrumentation such as nanomachines, robotics, nanomedicine, and diagnostic devices, among many others, will bring grand biomedical miracles to the world of medicine and other industries [3,4,5,6,7].

Nanoscale pertains to the size of one-billionth or 10^−9^ m of a material. A new scientific field of science in the form of nanotechnology was created because it was observed that materials, products, and devices developed from nanoscale particles almost always exhibit properties different from those of large-scale bulk materials. This follows the basic principles of physics and chemistry that as the state of matter is composed of atoms, any changes in atomic size, shape, and arrangement directly affect the material’s properties [7,8]. Scientists think that nanotechnology is the future of science and thus they are looking forward to benefitting from the application of nanotechnology in almost every possible way. The unique properties and behavioral features of nanoscale products have also drawn the attention of clinicians, physicians, and biological researchers [9,10]. The effort is on its way to applying unique quantum phenomena at the nanoscale to the fields of medicine, biomedical sciences, bioengineering, food technology, biochemistry, biophysics, and other disciplines of biology and medicine [10,11,12,13].

Forty years of revolutionary interaction among biology, medicine, and nanotechnology have led to present-day nano-biotechnology, which is now showing progressive application in multiple aspects of the medical field [14]. From disease detection to treatment, many medical issues such as disease diagnosis, drug discovery, personalized medical procedures, cancer treatment, pharmaceutical discoveries, as well as the latest medical tools and procedures, are now improving on the uses of nano-biotechnology [15]. Similar to regular vaccination approval, nano-based medicine and nanovaccines are also obtaining regular medical approval with the passage of time. Various nanotechnology-based diagnostic kits such as nanosensors, nanoparticle-based imaging agents, nanoparticle-based PCR Assays, Lab-on-a-Chip devices, along with modern drugs and medicines such as nanoparticle-based drug delivery vehicles, liposomal formulations and polymeric nanoparticles, Nanomedicines (such as Abraxane (nanoparticle albumin-bound paclitaxel) and Doxil (pegylated liposomal doxorubicin)), nanotechnology in gene therapy, nanoparticle-based vaccines, and antimicrobial agents, etc. are being commercialized for research and clinical usage [16].

Nanomedicine is a broad-spectrum field of science and technology that unites multiple streams of medical applications such as disease treatment and diagnosis, disease prevention, pain relieving technologies, human health improvement medicine, nanoscale technology against traumatic injury, and treatment options for diseases [12,15]. Thus, an interdisciplinary approach is being adopted to apply the outcomes of biotechnology, nanomaterials, biomedical robotics, and genetic engineering combined under the broad category of nanomedicine [17]. On a broader level, nanoscaling of medical technologies provides efficiency, a rapid response rate, and functional effectiveness in most biological and chemical processes used to manufacture medical materials. Thus, research provides constant hope for the upcoming new applications of nanomedicine [12,18].

In this review article, comprehensive analyses have been carried out to examine the application of nanotechnology specifically in the field of medicine. The most advanced form of nanotechnological applications have been highlighted with a slight emphasis on the previous uses of nanotechnology in the past few years of the 21st-century. Some modern medical applications, such as diagnostics, nanomedicine, regenerative medicine, and personalized targeted therapies, have also been included to bring into account the latest nanomedical applications.

## 2. Results and Discussion—Applications of Nanotechnology in the Medical Field

### 2.1. Applications of Nanotechnology in Diagnostics

Diagnostic sciences are now using nanodevices for early and rapid disease identification for further medical procedural recommendations. It also utilizes nanotechnology for the predisposition of disease at the cellular and molecular level to develop insights into treatment options [16]. Nanotechnology has the potential to revolutionize the field of healthcare diagnostics by improving the accuracy, sensitivity, and speed of medical tests [18]. One of the profound applications includes nanoparticle-based diagnostic imaging, in which nanoparticles can be attached to specific biomarkers to enhance imaging modalities such as magnetic resonance imaging (MRI), computerized tomography (CT) scans, and positron emission tomography (PET) scans, making them more sensitive, accurate, and specific [19]. Similarly, nanotechnology-enabled point-of-care diagnostic tests can quickly and accurately detect infectious diseases, cancers, and other illnesses, enabling timely treatment and prevention [9,19].

Biosensors are yet another dimension of application in which nanotechnology has enabled the development of highly sensitive biosensors that can detect even low levels of biomolecules in bodily fluids such as blood and urine, facilitating early detection and disease management [20,21]. Similar applications come in the form of microfluidic devices that incorporate nanomaterials and can be used to isolate and analyze specific cells, proteins, and genetic material, providing rapid and accurate diagnosis of diseases [19,22]. Another use may involve nanopore sequencing, which is a novel technology that uses nanopores to detect the sequence of DNA or RNA molecules, allowing for rapid and accurate diagnosis of genetic disorders such as cancer and genetic diseases [23].

Recent advances show that nanomedicine can be used in in vitro diagnostics sciences to increase the efficiency and reliability of disease apprehension [24]. This is achieved via nanodevices at the subcellular level, with samples prepared from human tissue, cell culture, body fluids, etc. [19,25,26]. In in vivo diagnostics, the nanomedicine approach is being used to develop devices capable of working, responding, and modifying within the human body with the sole purpose of early diagnosis of any irregularities in the human body that could lead to toxicity or tumor development events [22,27]. A few types of nanoparticles that are currently in use for diagnostic purposes include paramagnetic nanoparticles, nanocrystals, quantum dots, nanoshells, and nanosomes [28,29]. Overall, nanotechnology has enormous potential in healthcare diagnostics and is expected to play a significant role in the development of personalized medicine.

### 2.2. Nanotechnology and Lab-on-Chip Technology

Nanotechnology and Lab-on-Chip Technology have revolutionized the field of healthcare by offering innovative solutions for disease diagnosis, personalized treatment, and drug delivery [15]. The combination of these two technologies has led to the development of advanced diagnostic tools that are faster, more accurate, and more cost-effective than traditional diagnostic methods [30]. Lab-on-Chip technology is making progress in different fields of science; for example, it is being considered for use against viral and cancerous diseases [15,24]. The whole process revolves around analyzing genetic information at the cellular level [30]. Advanced procedures of gene sequencing and body fluid sampling have further assisted in revolutionizing nanotechnology in service of cures for diseases that were previously unimaginable [31,32].

Together, these two technologies have led to the development of Lab-on-Nanoparticles, which are small devices that can perform multiple functions, including diagnostics, drug delivery, and monitoring of various health conditions [31,32]. These devices are made up of nanoscale materials that can detect and respond to changes in the body, allowing for real-time monitoring and personalized treatment [26]. One of the significant applications of nanotechnology and Lab-on-Chip Technology in healthcare is cancer diagnosis [20,21]. Nanoparticles can be designed to target cancer cells, allowing for early detection and treatment [33]. Lab-on-Chip devices can also be used to diagnose various health conditions, including infectious diseases, genetic disorders, and metabolic disorders [32,34].

The use of nanotechnology and Lab-on-Chip Technology in healthcare has also led to the development of advanced drug delivery systems [31]. Nanotech systems such as nano-Liposomes can target specific cells or tissues in the body, enhancing drug efficacy and reducing side effects [28,35]. Moreover, viral detection is considered a feature that will be linked to future generations of nanoscale diagnostic devices. Such devices are expected to enable the detection of the release of medications in the organs of the body, which will help in the calculation of treatment efficiency and efficiency rates [36]. In simple terms, nanotechnology is trying to increase the pharmacokinetic and pharmacodynamic properties of drugs to stay longer inside the body, work faster and more efficiently, and at essential sites [37].

### 2.3. Applications of Nanotechnology in Pharmaceutical Sciences

**Figure 1:**
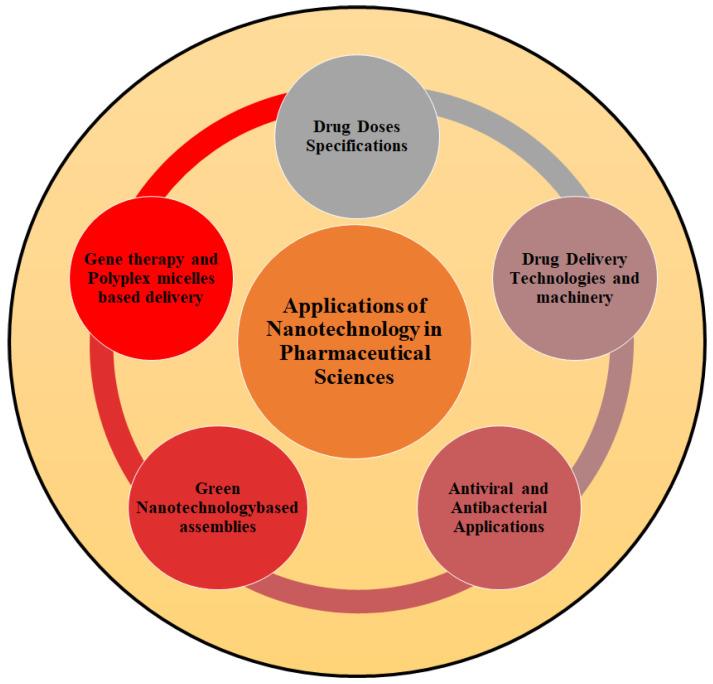
Applications of nanotechnology in pharmaceutical sciences.

A brief overview of nanotechnological applications in pharmaceutical sciences has been covered in the following section with a diagrammatic representation in Figure 1.

#### 2.3.1. Nanoscience and Drug Dose Specifications

Nanoscience has revolutionized the pharmaceutical industry by enabling the production of improved therapeutic drugs with enhanced efficacy and lower toxicity. Nanoparticles can improve the pharmacokinetics of drugs by increasing their solubility, stability, and bioavailability [38]. They can also target specific tissues and cells, reducing side effects and enhancing their efficacy [25]. The nanoscale size and unique physicochemical properties of nanoparticles demand precise specifications in terms of drug dose and administration [39,40]. The dose of nanoparticles depends on various factors such as their size, shape, surface properties, and the method of administration [40]. For instance, oral administration may require a higher dose to achieve the same effect as intravenous administration due to the differences in absorption and biodistribution [40,41].

Furthermore, nanoparticles have complex pharmacokinetics and dynamic behavior in vivo, requiring a careful consideration of their dose regimen [40]. Researchers need to determine the optimal dose range, frequency, and duration of nanoparticles to achieve their therapeutic goals while minimizing adverse effects [41,42]. In the past, medical studies have resulted in very advanced treatment options; however, there is still a gap in effectively neutralizing drug overdoses. The use of nanoparticles as absorbents of toxic drugs is a feature being taken into account to create a rich method of drug absorption in the medical sciences [40,41,42]. The design of nanosponge-type substances is on the way to absorb unnecessary toxic dosages of drugs in blood to reduce the side effects of drug overdoses and treat ailments from body fluids [43]. Such antiviral drug absorbents have been introduced by researchers that work as nanoscale molecules to render anticancer and antiviral nucleoside analogs by linkage with squalene [44]. These nano-assemblies work as superior anti-cancerous molecules to treat human cancer cells that have yet to be developed beyond in vitro studies [45]. In summary, the development of nanomedical products requires careful consideration of the dose and administration of nanoparticles to ensure their efficacy and safety. The nanoscience community must collaborate with regulatory agencies to develop guidelines for nanomedicine testing to ensure their safety and efficacy.

#### 2.3.2. Nanotechnology and Drug Delivery Technologies

Nanotechnology has revolutionized the field of drug delivery by providing an effective and targeted delivery of drugs, minimizing side effects, and increasing the therapeutic efficacy of drugs. The application of nanotechnology in drug delivery involves the use of nanoparticles that are designed to carry drugs and deliver them to the desired site of action [46]. The use of nanotechnology in drug delivery has several advantages. First, it allows for targeted and controlled delivery of drugs to specific sites in the body, such as tumors, inflamed tissue, and infected areas [46]. This reduces the amount of drugs required and minimizes side effects. Secondly, nanoparticles can improve the solubility and stability of drugs, making them more effective in treating diseases [47]. Thirdly, nanotechnology can increase the bioavailability of drugs by enhancing their absorption and distribution in the body. This allows for lower doses of drugs to be used, resulting in reduced toxicity [48,49].

Drug delivery technologies are also being given full consideration to be modified as per the new rules of nanoscaling. Some kinds of medical nanorobots are in line to be used for medicine delivery [32]. These materials swim across veins and carry drugs to specific sites. These aspects are being used for antitumoral responses of drugs [48]. Scientists are even working on performing wireless intracellular and intranuclear nanoscale surgeries against multiple malignancies and diseases [46,48]. Marvelous scientific arrangements are being carried out in the form of manufacturing and testing mechanical red blood cell technologies called respirocytes. Nanorobotics share the potential to deliver 200+ times more oxygen to body tissues as compared to natural red blood cells [49,50]. This could make one think about the potential of nanotechnology to be utilized for the diagnosis and treatment of various blood-linked disorders and their cure in the future [50]. In conclusion, the application of nanotechnology in drug delivery has revolutionized the field of medicine. It has provided an effective and targeted delivery of drugs, minimized side effects, and increased the therapeutic efficacy of drugs. The future of drug delivery lies in the continued development of nanotechnology-based drug delivery systems.

#### 2.3.3. DNA Nanotechnology and Drug Delivery System

DNA-based drug delivery devices have been introduced in the past few years, such as DNA guns and DNA vaccines. Based on similar principles, an emerging field of DNA nanotechnology is being introduced in the nanomedicine industry [51]. These medical tools allow for the self-assembly of nanostructures and molecules that ultimately enhance drug targeting and reduce the toxicity associated with these drugs. With such technology, toxicity measures can be easily dealt with in diseases such as cancer, where the major issue is the drug toxicity associated with chemotherapeutic drugs [24,51].

The latest advances in research indicate that modern programing optimization and in silico approaches are being adopted to design DNA nanostructures with precise size, structure, surface chemistry, and functioning properties against specific diseases [22,52]. The effort is also to create personalized targeted drug therapies using nanotechnology-based DNA medicine [51]. Efficient drug biomolecules, such as doxorubicin and CpG oligonucleotides, have been successfully amalgamated with DNA-based nanostructures to increase cellular intake efficiency [53]. The future holds the potential to create RNA-based medication using principles similar to those employed in DNA-based medication [54].

#### 2.3.4. Nanobiotechnology and Gene Therapy

Nanobiotechnology and gene therapy are two fields that often intersect in the development of innovative therapeutic approaches for the treatment of various diseases. In gene therapy, DNA molecules are introduced into the patient’s cells to replace defective or missing genes, with the aim of treating genetic disorders and other diseases [55]. One application of nanobiotechnology in gene therapy is the use of nanoparticle-based delivery systems to transport therapeutic genes to target cells [41,56]. These nanocarriers protect the DNA molecules from degradation and enhance their ability to penetrate the cell membrane, increasing the efficacy and safety of gene therapy [53,56].

Other nanobiotechnology approaches that support gene therapy include the development of gene editing technologies that use nanoscale tools to precisely modify DNA sequences and correct genetic mutations [57]. Additionally, nanoparticle-based sensors can be used to monitor gene expression and other molecular events in real-time, providing valuable information for personalized medicine [32]. Modern therapeutic concepts including gene therapy and molecular DNA-based therapies are already being practiced in healthcare and the arrival of nanotechnology has forwarded further advances in it [58]. Since the very basis of working gene therapy is at the molecular level of disease prevention and genetic adjustments, nanoscale technology plays a vital role in gene therapy [58].

Gene therapy processes are being modified to attach different kinds of biodegradable and non-biodegradable organic and inorganic particles fabricated with nano-assemblies. These structural combinations help bind DNA and access it across cellular surfaces [59]. Moreover, polymer-based nanoparticle mixtures are also prepared for intravenous drug injections. These modified technologies are a gateway to further advances in nanogenetic therapies [60]. Overall, the integration of nanobiotechnology and gene therapy is expected to lead to advanced treatments for a wide range of diseases, including cancer, genetic disorders, and infectious diseases.

##### Gene Therapy Approaches via Polyplex Micelles

Polyplex micelles are a type of nano-sized structure that are formed by the self-assembly of cationic polymers with nucleic acids, such as small interfering RNA (siRNA) or plasmid DNA (pDNA) [61]. These polyplex micelles have attracted significant attention for their potential in gene therapy and as drug delivery systems. In the context of tumor treatment, various polyplex micelle-based strategies using siRNA and pDNA have been studied. siRNA is an RNA molecule that is used to specifically target and knock down the expression of disease-related genes [62,63]. Plasmid DNA (pDNA) is a circular DNA molecule that can carry therapeutic genes to the target site. Polyplex micelles can encapsulate siRNA or pDNA within their core, protecting them from degradation and facilitating their delivery to tumor cells [63,64]. Additionally, the cationic nature of the polyplex micelles allows for electrostatic interactions with the negatively charged cell membrane, promoting their uptake by tumor cells [64].

These polyplex micelle-based strategies have been investigated for the treatment of various tumors, including pancreatic adenocarcinoma [63]. Pancreatic adenocarcinoma is a particularly challenging type of solid tumor resistant to many conventional treatment options. By using polyplex micelles, siRNA or pDNA can be delivered specifically to the tumor cells, enabling targeted gene therapy or enhancing the efficacy of chemotherapeutic drugs [64]. Thus, nanotechnology, specifically polyplex micelles, offers a promising approach for delivery of siRNA or pDNA to tumors such as pancreatic adenocarcinoma. These micelles can protect genetic material, promote cellular uptake, and potentially enhance the effectiveness of treatments for intractable solid tumors [65].

#### 2.3.5. Green Nanotechnology-Driven Drug Delivery Assemblies

Nanomedicines are largely produced through chemical and physical methods of downgrading particles up to micro- and nanoscales. However, with the concerns of environmental and toxic health impacts, nanomedicine is now employing the concept of green chemistry and green engineering into the manufacturing of nanobiomedicine [66]. The purpose of this green technology is to create eco-friendly nanoassemblies with less environmental and health-related negative impacts [66]. Subsequently, the combination of green nanoassemblies with drugs, vaccines, or diagnostic markers will be the next step to propel the field of green nanomedicine. Many inorganic nanoassemblies have been introduced to the market and manufactured on the principles of green engineering and nanotechnology [67]. Some examples may include gold and silver nanoparticles, quantum dots, organic polymeric nanoparticles, mesoporous silica nanoparticles, dendrimers, nanostructured lipid carriers, solid lipid nanoparticles, etc. [66,67,68].

These nanoassemblies are attached with drugs, DNA molecules, or specific enzymes, proteins or peptides for further handling in nanomedicine purposes [66]. However, the need is to establish research studies that demonstrate the difference and effectiveness level of nanomedicine produced using normal bioengineering against that of manufacturing of nanomedicines through the elaborative principles of green bioengineering [66,67,68]. This will allow scientists to opt for the best manufacturing conditions for nanoassemblies in the future.

#### 2.3.6. Nanotechnology—Antiviral and Antibacterial Applications

The causative agents of viral, bacterial, and other microscopic diseases work at the microscopic level; therefore, the best way to fight against them is at the nanoscale. Nanotechnology is thus the gateway to the cure and diagnosis of a wide range of viral, bacterial, and fungal diseases [69]. Although traditional Greek medicinal practices have been using metals such as silver to cure diseases for a long time, an updated version of nanoscale-based material conversion has been shown to improve the efficiency of such traditional and modern medication options [70]. One such study carried out by Nycryst Pharmaceuticals (Canada) showed that nanosized silver particles are more reactive to cure burn or wound as they easily penetrate the skin at some small scale [71].

The genomic and proteomic fields are already contributing much to the elucidation of molecular insights into disease, and with the assistance of nanotechnology, new opportunities are being put in the hands of researchers to create powerful diagnostics tools with the power of genetic elucidation of irregularities at the level of the gene [72]. Research indicates that soon, nanotechnology-based diagnostic and treatment options will be available for preventive and regenerative medicine with targeted and personalized therapy potential against pathogenic and pathophysiological diseases [70,71,72,73]. All these benefits are coupled with the cost-effective and time-saving aspect of this new technology.

#### 2.3.7. Barriers Associated with Nanoparticle-Based Delivery Efficiency and Clinical Translation

There are several barriers or issues associated with nanoparticles in terms of delivery efficiency and clinical translation. The accumulation of nanocarriers in organs of the reticuloendothelial system, especially the liver, poses a significant challenge for clinical translation as it captures a majority of the injected dose, hindering the delivery of an adequate dose to the targeted disease site and potentially causing toxicity concerns [74]. Researchers have developed various approaches to address this issue, including preconditioning macrophages with chloroquine, saturating the reticuloendothelial system organs with drug-free nanocarriers, and transient stealth-coating scavenger cells to enhance the efficiency of drug-loaded nanoparticles reaching the diseased tissue [75,76,77]. Additionally, the incorporation of targeted cellular on the surface of nanocarriers such as those applying the “do not eat us” strategy, helps evade capture by the reticuloendothelial system, improving the accumulation of nanodrugs at the desired site [73,76].

On the other hand, surface shielding of nonionic hydrophilic polymers such as PEG on nanocarriers reduces cellular uptake and endosomal escape, resulting in poor delivery efficiency despite improving colloidal stability and stealth in a biological environment [77]. To overcome this “stealth dilemma,” targeting ligands are strategically placed at the distal end of the PEG segments to facilitate specific ligand receptor-mediated uptake [78]. Another strategy involves wrapping anionically charged polymers on positively charged mRNA-polyplexes to promote endosomal escape by converting them into positively charged polymers in response to the acidic pH of the endo/lysosomal compartments [79].

The use of messenger RNA (mRNA)-loaded lipid nanoparticles is limited by their hepatic protein expression, even when administered locally through intramuscular and intratumor injections [80]. Minimizing the off-target hepatic expression would be advantageous for protein replacement therapies and cancer immunotherapies. One approach involves incorporating microRNA target sites in therapeutic mRNAs to selectively prevent their expression in the liver [80]. Some other generalized barriers associated with nano-based drug delivery mechanisms are included in Table 1. It is important to note that although nanoparticles face these barriers and issues, significant advancements are being made in addressing them, bringing us closer to their successful clinical translation.

### 2.4. Applications of Nanotechnology in Regenerative Medical Sciences

#### 2.4.1. Nanotechnology and Bone Regeneration Technology

Nanotechnology is the science of creating and manipulating materials at the molecular and atomic levels. Bone regeneration technology creates new bone tissue, or helps existing bone tissue heal, with the use of materials that promote bone growth [81]. Nanotechnology is increasingly used in bone regeneration technology to create better, more precise and targeted materials for promoting bone growth [80]. For example, researchers are exploring the use of nanoparticles to deliver drugs or other molecules that promote bone growth directly to the areas that need them, improving the effectiveness of the treatment [80].

Nanoparticles can also be used to create scaffolds that mimic the structure of bone, which can help guide new bone growth and aid in bone regeneration. Additionally, advances in 3D printing technology that uses nanoscale materials can be used to create highly precise and customized implants for bone regeneration [81]. Bone weakening and dysfunction is a widespread problem and this has been marked by nanotechnologists as an issue of the utmost importance when linking nanotech to medicine. Some studies are being carried out regarding bone formation and structuring with the help of nanotechnology [80,81]. Scientists are trying to develop bone graft substitutes in the form of nanostructured materials with similar properties to be accepted by body and organ tissues. If these studies succeed, they will bring a new wave of regenerative technology to cure damaged bones and broken muscular fragments [82].

Principle investigation on biomineralization is being carried out to reduce the particle size of bone materials that could be coupled with its crystalline properties to be embedded into collagen fibers [80]. The purpose is to create a penetrating composition in damaged bone areas with specific mechanical properties to revolutionize the field of osteology and bone tissue engineering [80,81]. Similar studies are being carried out to make artificial joints, nanoscale collagen-mimicking coatings for knees and hips that act to stabilize the process of bone formation by osteoblasts [83,84]. Overall, the use of nanotechnology in bone regeneration technology holds great promise for improving the outcomes of bone repair and regeneration, including faster healing times, improved bone strength, and reduced complications.

#### 2.4.2. Nanotechnology and Regenerative Medicine

Regenerative medicine is an interdisciplinary field of medical applications in which the benefits of cell therapy and tissue engineering methods are well fabricated to device mechanisms for the treatment, maintenance, improvement, and reparation of damaged and dead cells, tissues, and organs [73]. Previously, it was difficult to deal with the body at the cellular level but with the emergence of nanoscale technology, a huge opportunity has become available in the form of regenerative medicine to interact with cells and their components so that the linked cellular responses and extracellular material production can be controlled [80]. Tissue repair has been greatly upgraded with the powerful tissue regeneration abilities of nanoassemblies. These technologies are being directed for cellular adhesion, migration, differentiation, and other mechanical aspects that initiate tissue regeneration [85].

Exploration in the field of nanomedicine is going on to manufacture nanoscale materials, such as gold and silver nanoparticles, dendrimers, nanorods, carbon buckyballs, nanoshells, nanocubes, and many other forms of nanoparticles [73,79]. Each is specific to its linked properties, which can be directly utilized in targeted tissues and organs. Multiple research groups are working worldwide to explore the diagnostic, therapeutic, anti-viral, antifungal, and most importantly anticancerous properties of these nano-agents [70,72,86]. Progress shows that soon, a world of nanotechnology will bring a revolution to the treatment options for incurable diseases such as cancers, for which early diagnosis through nanotechnology is already on board and has been successfully explored [73,86].

### 2.5. Applications of Nanotechnology in Surgery

**Figure 2:**
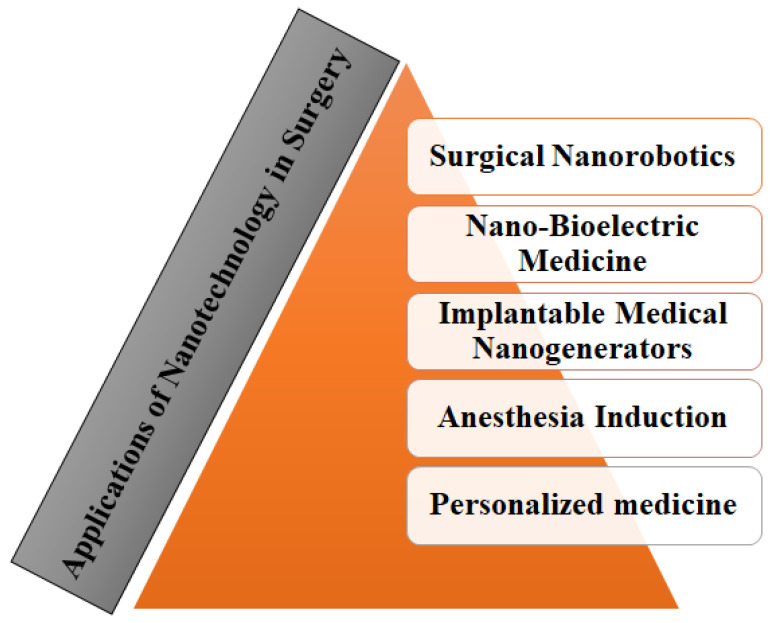
Applications of nanotechnology in surgery.

A brief overview of nanotechnological applications in surgery is covered in the following section with a diagrammatic representation in Figure 2.

#### 2.5.1. Surgical Nanorobotics and Nano-Bioelectric Medicine

Surgical nanorobotics involves the development and use of tiny robots or nanorobots that can perform surgical procedures with high precision and efficiency [87]. These nanorobots can be guided to specific locations within the body using advanced imaging techniques, and they can then perform tasks such as delivering drugs, removing tumors, or repairing damaged tissues. Nano-bioelectric medicine, on the other hand, involves using electrical signals to stimulate the body’s healing processes [88,89]. This emerging field focuses on the use of nanoscale technologies to access and control the electrical activity of cells and tissues in order to treat a wide range of medical conditions, including chronic pain, wound healing, and heart disease [90]. Both surgical nanorobotics and nanobioelectric medicine have the potential to revolutionize the field of medicine and improve patient outcomes. However, there is still much research needed to fully explore the potential of these technologies and ensure their safety and efficacy [88].

Programming, engineering, and biological fields are working inter-connectively to develop a surgical nanorobot that works through the vascular system. These small-scale devices are manufactured with the multipurpose function of searching diagnostics and treatments against lesions and pathogens [87,88]. These robots work at a minute scale that can be used to cut even a single dendrite and neuron at the cellular surgery level without causing harm to other neurons bound in a complex network. These experiments have been confirmed in animal models where a nanoscissor action has been governed by these nanorobotics [91]. The results have pushed scientists to perform further experiments before optimizing surgical conditions on diseased patients. A new wave of bioelectric medicine is also in the market which adheres to biological components for more effective diagnostic and therapeutic therapies. This nanobioelectronic is being employed in cancerous diseases, cardiovascular disorders, and other malfunctions in the human body [92]. However, many improvements are needed to successfully apply this technology in a clinical setting for multipronged complex diseases.

#### 2.5.2. Implantable Medical Nanogenerators

Nanogenerators, as the name indicates, are a class of self-powered and implantable medical nanosensors. They work on the principle of conversion of mechanical energy from body movement into an electric spark [87]. As the body converts chemical energy from glucose, muscle converts this energy to mechanical energy and in turn these nanogenerators convert it into electric energy which can be used to charge and power implantable nanodevices that are aggressively being manufactured for medical purposes nowadays [88]. Implantable medical nanogenerators (IMNGs) are miniature devices that use mechanical energy from body movements to generate electrical energy [87]. They can be implanted inside the human body and used to power various medical devices, including pacemakers, neurostimulators, and drug delivery systems [93].

IMNGs are made up of thin layers of materials, such as piezoelectric materials, which convert mechanical energy into electrical energy. These materials generate electric charges when they experience mechanical stress, such as bending or pressure [87]. They can also be designed to harvest energy from other sources, such as temperature changes or fluids in the body [88]. IMNGs have several advantages over traditional batteries used to power implantable medical devices. They can eliminate the need for battery replacements, which can be invasive and costly. They can also improve device reliability as battery failures can cause serious medical problems [93]. Additionally, IMNGs are environmentally friendly since they do not require the disposal of toxic batteries [94].

Despite their potential benefits, there are still challenges to overcome in developing IMNGs. The devices must be durable enough to withstand the harsh conditions inside the body, including high temperatures and corrosion from body fluids [95]. They must also be small enough to be implanted inside the body without causing discomfort or obstruction [94,95]. Overall, IMNGs hold great promise for improving the safety, reliability, and convenience of implantable medical devices in the future. Therefore, researchers are continuously working toward their development to make them practical for human use.

#### 2.5.3. Nanotechnology and Anesthesia Induction

Anesthesia induction is a critical step in dental surgeries and other sensitive medical procedures, such as brain surgeries. For such anesthesia induction procedures, researchers are working on nanorobotic suspension mixtures that make a colloidal suspension with millions of nanoscale active analgesic nanoparticles [96]. These nanoparticles work on patients’ gingival and other sensitive portions and penetrate deep up to the level of loose tissue. This passage of nanomaterials is conducted via the combinational principles of chemical and temperature gradients and positional navigation that are monitored and controlled by onsite nanocomputers [97]. This nanoscale anesthetic action helps to carry out the desired effect, attained quickly with an even distribution of anesthetic in the projected organ such as the dental surface. The sensitivity action can also be controlled for a particular tooth for which surgical action is required. After the completion of surgeries, nanorobots are controlled via nanocomputers to restore tooth sensitivity to normal [98].

### 2.6. Applications of Nanotechnology in Dentistry

Nanodentistry is a separate branch of nanomedicine that involves a broad range of applications of nanotechnology ranging from detection to diagnosis, to cure treatment options and prognostic details about tooth functions [99]. A wide spectrum of oral health-related issues can be dealt with using nanomaterials [100]. These nanomaterials derive their roots from tissue engineering and biotechnologically manufactured dental nanorobotics [100,101]. Some recent advances under oral nanotechnology may include treatment options such as anesthesia, dentition renaturalization, hypersensitivity cures, orthodontic realignment problems, and modernized enameling options for the maintenance of oral health [99,102].

**Figure 3:**
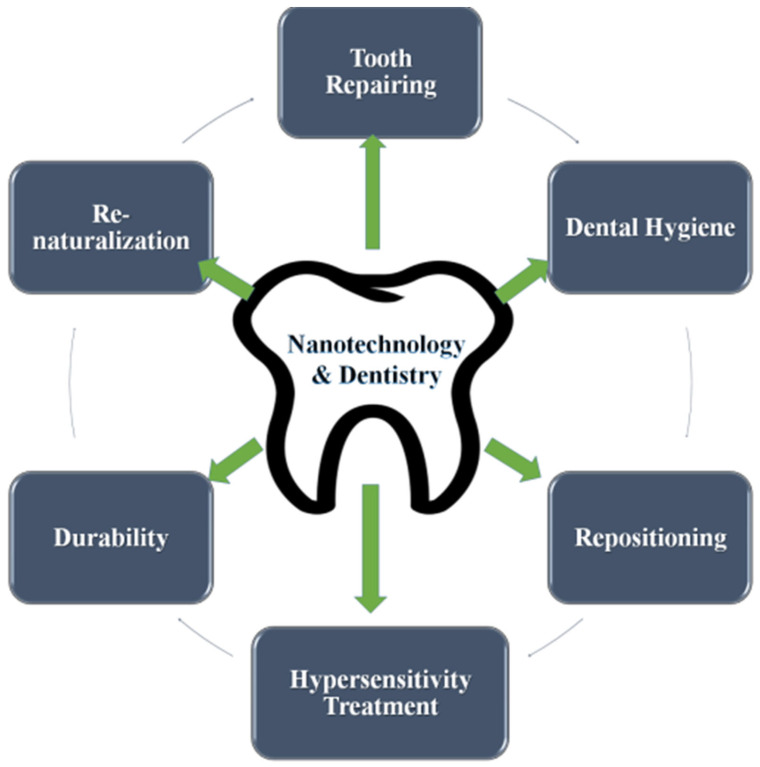
Major applications of nano-dentistry.

The nanoscale technology used for such functions are named mechanical dentifrobots. They work to sensitize nerve impulse traffic at the core of the tooth in real-time calculation and hence could regulate the tooth tissue penetration and maintenance for normal functioning [103]. The functioning is coupled with programmed nanocomputers to execute actions from external stimuli via connection with the localized internal nerve stimuli. These mechanistic insights could help dental surgeons suggest a strategic treatment option that may be conducted directly via in vivo nanorobot action using acoustic signals, as elaborated earlier [100,101,102,103,104]. Some of the applications of nanotechnology in the field of dental science have been compiled at the end of this section in Figure 3.

#### 2.6.1. Nanotechnologies, Tooth Repair, and Hypersensitivity Treatment

Scientists are further working to use nanotechnology for the creation of dental cures and treatment strategies. This may include the stimulation of the natural biomineralization process or the utilization of nanomaterials for artificial tooth development with sensitivity programed by nanorobotics [100,105]. They are trying to develop the hardest tissue enamel by using nanoscale manufacturing of nanorods derived from calcium hydroxyapatite crystals to help regulate the function of teeth. Additionally, reconstructive dental nanoparticles are utilized to offer patients a rapid and long-term cure against hypersensitivity [106].

#### 2.6.2. Tooth Repositioning and Renaturalization

Repositioning of the tooth is a matter of greater concern for patients as it sets the basis for further cure or disruption of dental health in case of maladjustment. Orthodontic nanorobots could be used in this case to manipulate tissues in such a way that a smooth painless straightening, rotation, and repositioning of the tooth could be attained [107]. Moreover, with time, customers are more interested in improving the aesthetic standing of their physical appearance, and so the concept of dental esthetics has emerged. In this regard, nanotechnology is considered to perform actions such as excavating dental amalgams or remanufacturing teeth alongside fillings, crowns, and other such modifications [107,108].

#### 2.6.3. Nanotechnology and Dental Durability

Much more effort is being put into securing dental durability and the appearance of teeth in normal dentistry practices. Nanotechnology provides a more secure and long-lasting solution in the form of nanostructured dental materials with carbon nanotubes that provide fracture-resistant properties [109]. Additionally, simpler dentifrobots are being incorporated into mouthwashes and toothpastes to replenish dental surfaces on a routine basis for cleaning and continuous calculus debridement [110]. These dentifrobots have the ability to highlight and destroy specific pathogenic bacteria from the mouth and retain the useful oral microflora in a healthy balance [111]. All these benefits delay the conventional causes and processes of dental decay with the remedial disappearance of oral diseases, especially in the early years [100,112].

### 2.7. Applications of Nanotechnology in Oncology Field

#### 2.7.1. Nanotechnology and Cancer Treatment Strategies

In the world of medicine, complex and incurable diseases such as cancer are always given a special focus to find treatment and early diagnosis options for these modalities [113]. Nanotechnology is providing a good opportunity for researchers to develop such nano-agents, fluorescent materials, molecular diagnostics kits, and specific targeted drugs that may help to diagnose and cure disease in a better way in the future [114]. Scientists are trying various protocols to conjugate already available drugs with nanoparticles to enhance drug specificity and targeting in organs [113,114,115].

Nanomedicine acts as the carrier for hundreds of specific anticancerous molecules that could be projected at tumor sites. Moreover, the tumor imaging and immunotherapy approaches linked with nanomedicine must also be kept in mind when diving deep into nanomedicine and cancer links [34]. The effectiveness of nanomaterials in cancer therapies has pushed scientists to replace traditional cancer therapy approaches with targeted therapies that may be utilized alone or in conjugation with already available anti-cancerous drugs [16,34]. The focus is also being drawn toward lessening the impact of chemotherapeutic drugs by increasing their tumor-targeting efficiency and improving their pharmacokinetic and pharmacodynamic properties. Similarly, heat-induced ablation treatment against cancer cells alongside gene therapy protocols are also being coupled with nanorobotics [52].

**Figure 4:**
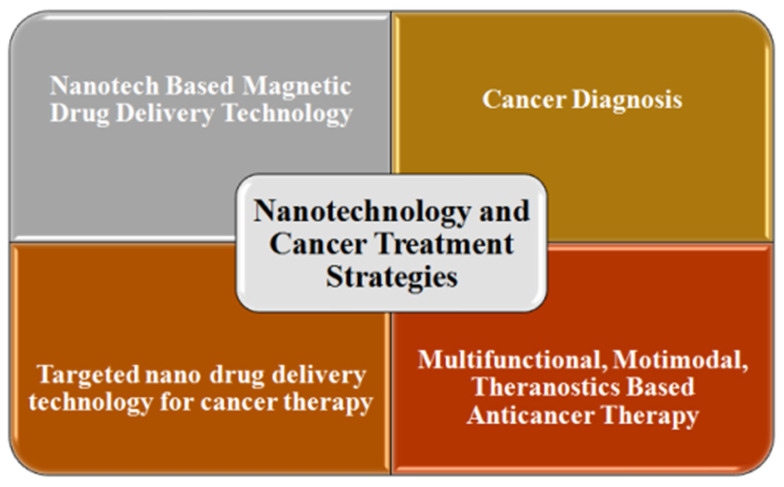
Applications of Nanotechnology in Oncology field.

Some other cancer treatment options, in the form of enhanced tissue imaging and tumor microenvironments, as well as adjustment by the release of nanoparticle-bounded drugs, are being practiced in the oncology field [59,116]. These nanomedicines hold the potential to overcome drug solubility, instability, and resistance issues. Various nanomedicines that act as anticancerous medicines are being researched, while some have been approved by the US Food and Drug Administration (FDA) and European Medicine Agency (EMA) [117]. These anticancerous drugs may utilize the “Enhanced Permeation and Retention Effect” (EPR effect) and/or active targeting of nano assemblies such as liposomes, albumin nanospheres, micelles, and gold nanoparticles [118]. Some of the applications of nanotechnology in the oncology field are discussed in the following section and a summary (Figure 4) is shown at the end of this section.

#### 2.7.2. Nanotechnology in Cancer Diagnosis

Cancer diagnosis is the most observable problem in cancer patients. Cancer largely remains uncured due to late detection in the third or fourth stages. To fight this cause, nanotechnology is being employed to allow early detection of tumors in organs [16]. Nanotechnology provides a very sensitive and specific multiplexed measurement capacity to detect cancer biomarkers in extracellular settings and in vivo bioimaging techniques [19]. Nanotechnology has enormous potential in the field of cancer diagnosis. Nanoparticles are incredibly small and can penetrate cell walls and the blood–brain barrier. This makes them ideal for delivering drugs and other therapeutic agents to cancer cells. They can also be used to detect cancer cells and identify the location and nature of the disease [119].

One of the most promising areas of nanotechnology in cancer diagnosis is the development of targeted nanoparticles. These are nanoparticles designed to adhere specifically to cancer cells, allowing them to be easily identified and targeted by doctors. This could result in more accurate early detection, better monitoring of cancer progression, and faster diagnosis [120]. Another promising application of nanotechnology in cancer diagnosis is in the development of biosensors. Biosensors are small devices that can detect specific biomarkers in a patient’s blood or other bodily fluids. These biomarkers can be indicative of cancer and could be used to detect cancer at an early stage [92,93].

In conclusion, nanotechnology has enormous potential in the field of cancer diagnosis. With targeted nanoparticles and biosensors, it could help in the development of a more accurate, non-invasive and effective way to diagnose cancer. However, the challenges pertaining to such diagnostic kits remain and the need is to overcome these challenges and update the nanotechnology-based diagnostic methods for cancer and other disease diagnostics and prognoses in the future [19,120].

#### 2.7.3. Multifunctional, Multimodal, Theranostics-Based Anticancer Therapy

Multifunctional theranostics therapy is an emerging field in cancer treatment that combines multiple modalities into a single treatment approach. This approach aims to both diagnose and treat cancer using nanomaterials. Nanomaterials, such as nanoparticles, are highly versatile due to their unique properties at the nanoscale [121]. They can be engineered to have various functionalities, such as imaging capabilities, drug delivery systems, and targeted therapy agents. By using these multifunctional nanomaterials, theranostics therapy can provide simultaneous cancer diagnosis and treatment [122]. In parallel, the term multimodal refers to the combination of multiple treatment modalities in a single therapy [123]. In the context of theranostics therapy, multimodal treatment can involve different approaches, such as chemotherapy, radiotherapy, and immunotherapy [124]. These modalities can be incorporated into nanomaterials used for therapy, allowing for targeted delivery and enhanced efficacy. The theranostic approach also enables real-time monitoring of treatment outcomes [124]. By incorporating imaging agents into nanomaterials, clinicians can track the distribution and effectiveness of the therapy. This information helps guide treatment decisions and allows for adjustments to optimize patient outcomes [123]. Thus, the combination of multifunctional and multimodal theranostics therapy using nanomaterials holds great promise in the fight against cancer. It offers the potential for personalized and targeted treatment, improved efficacy, and reduced side effects compared to traditional cancer therapies [121,125].

#### 2.7.4. Targeted Nano Drug Delivery Technology for Cancer Therapy

Targeted nano drug delivery technology for cancer therapy is a form of treatment that uses nano-sized particles to deliver drugs specifically to cancer cells in the body. These nanoparticles can be engineered to selectively bind to cancer cells, allowing the drugs to be delivered directly to the tumor site, while minimizing damage to healthy tissues [5]. The development of targeted nano drug delivery systems has several advantages in cancer therapy. Firstly, it can enhance the efficacy of the drugs by increasing their concentration at tumor sites. This is especially important for drugs with low solubility or high toxicity as it allows for higher doses to be delivered directly to the cancer cells [24]. Additionally, targeted nano drug delivery systems can help overcome some limitations of traditional chemotherapy, such as poor drug bioavailability or resistance. By encapsulating the drugs within nanoparticles, their stability and solubility can be improved, leading to better drug delivery and higher therapeutic effects [5,126].

There are various types of targeted nano drug delivery systems being explored, including liposomes, polymeric nanoparticles, dendrimers, and carbon nanotubes. These nanoparticles can be functionalized with ligands or antibodies that specifically bind to receptors or proteins overexpressed on the surface of cancer cells [127]. This targeting moiety allows for the selective binding and internalization of nanoparticles into cancer cells, enabling efficient drug delivery. Furthermore, targeted nano drug delivery systems can also be combined with imaging agents, enabling real-time monitoring of drug distribution, tumor targeting, and uptake [24,128]. This helps in tracking the therapeutic response and adjustment of treatment protocols as needed [128]. Overall, targeted nano drug delivery technology has the potential to revolutionize cancer therapy by improving the efficacy and safety of drugs, minimizing systemic side effects, and enabling personalized medicine approaches. However, further research and development is still needed to optimize these systems and ensure their clinical translation [128,129].

#### 2.7.5. Nanotech Based Magnetic Drug Delivery Technology and Cancer Therapy

Nanotechnology and magnetic drug delivery technology are both innovative approaches in the field of medicine that improve drug delivery and enhance treatment effectiveness. Magnetic drug delivery technology utilizes the application of an external magnetic field to guide drug-loaded nanoparticles to a specific site within the body [130]. Magnetic nanoparticles can be functionalized with drugs and then injected into the bloodstream. By applying a magnetic field externally, the nanoparticles can be directed toward the desired location, such as a tumor [131]. This approach allows for more precise drug delivery, minimizing systemic exposure and reducing side effects [39]. Similarly, by engineering nanoparticles, researchers can create drug carriers with unique properties that are not in conventional drug delivery systems [130,131,132]. These nanoparticles can be functionalized and designed specifically to target diseased cells or tissues, improving drug concentration at the desired site and minimizing off-target effects [131].

Additionally, nanoparticles can protect the drug payload from degradation, resulting in improved stability and prolonged drug release. Thus, the combination of nanotechnology and magnetic drug delivery technology has shown promise in several areas of medicine [130]. For example, in cancer treatment, magnetic nanoparticles can be used to deliver chemotherapy drugs directly to tumors, increasing drug concentration at the tumor site and reducing toxicity in healthy tissues. This approach can enhance treatment efficacy while minimizing adverse effects [133]. Furthermore, magnetic drug delivery can also be utilized in targeted therapy for other diseases, such as neurological disorders. Nanoparticles loaded with neuroactive drugs can be guided to specific regions in the brain using externally applied magnetic fields, allowing for more targeted treatment and potential reduction in systemic side effects [133]. Thus, the integration of nanotechnology and magnetic drug delivery technology has the potential to revolutionize drug delivery by improving targeting, reducing side effects, and enhancing treatment outcomes. Ongoing research and development in this field hold great promise for the future of medicine.

### 2.8. Other Applications of Nanotechnology in the Medical Field

#### 2.8.1. Applications of Nanotechnology in Medical Machinery

As nanotechnology is making progress in the field of medicine and biological sciences, eyes are on the board as to how this technology will bring revolution to medical machinery [25]. It is predicted that soon, micro and nanoscale materials will be integrated with useful robotic characteristics that may include nanoscale manipulator arms, sorting rotors, reagent purification kits, and super diagnostic surfaces that will be modeled to respond to particular disease diagnostics and treatment. These nanomaterials and robotic connections are assumed to be controlled via nanocomputers [25,134].

Nanocomputers are expected to control, activate, deactivate, and deter the response rates of nanomechanical devices [134]. They will be programed to execute specified medical and dental operations with a connection to a wider network of interconnected nanocomputers, such as programmed nanomachines and robotics, which have the potential to allow physicians and clinicians to perform precise medical procedures at a subcellular level [135,136,137]. Furthermore, these robotic elements are expected to work in gerontological and pharmaceutical research phases, diagnostics, and dentistry [138].

#### 2.8.2. Nanotechnology and Veterinary Medicine

In addition to the application of nanomedicine to humans, beneficial applications of nanomedicine are now being used on animals. Multiple variations of nanovaccines and nanoadjuvants have started their way into veterinary sciences [11,139]. The previously used animals’ therapeutic, diagnostic, treatment, and veterinary vaccinations along with disinfection, breeding, reproduction, and nutritional concerns are now being modernized using the concept of nanotechnology [139].

Nanotechnology has the potential to revolutionize the field of veterinary medicine, offering new diagnostic tools and treatment options for animals. In the area of diagnostics, nanotechnology can improve the accuracy and sensitivity of diagnostic tests used to detect various diseases [140]. Nanoparticles can be engineered to bind to specific biomarkers in the body that are indicative of disease, allowing for early detection and treatment [140]. In the field of therapeutics, nanotechnology can improve drug delivery systems, enhancing drug efficacy while minimizing side effects. Nanoparticles can be designed to improve drug solubility, stability, and specificity, ensuring that drugs reach their intended targets and remain active for longer periods of time [139,140,141,142].

Additionally, nanotechnology can be used to develop novel vaccines and immunotherapies, as well as new tools for regenerative medicine. For instance, nanoparticles can be used to create scaffolds for tissue engineering and repair, promoting the growth of new tissue and accelerating healing processes [73,85,86]. The use of such small-scale nanomedicine shows a direct impact on public health due to the interconnectedness among humans and animals within the same living environment. The effort is going on to increase meat and milk production, leading to a reduction in vaccine residues and drug resistance problems in veterinary medicine [142,143]. Moreover, this medicinal revolution remains cost-effective and helps to minimize the amount of discarded milk and meat products. In addition to that, in modern pet care, nutritional and hygienic products are also being introduced in the market under the genesis of successful practices in nanotechnology [143]. Overall, nanotechnology offers exciting possibilities for improving animal health and welfare and has the potential to revolutionize veterinary medicine.

#### 2.8.3. Nano Sensors, Nano Microbivores and Chemical Warfare Technology

Nanosensors refer to small devices that can detect and analyze chemical or biological agents at the molecular level. They have various applications, including monitoring air quality and detecting pathogens in food and water [12]. Nano-microbivors, on the other hand, are small (microscopic) organisms that can consume or break down contaminants such as organic chemicals and heavy metals in the environment [17]. They can be used for bioremediation purposes and for treating contaminated soil and groundwater [144,145]. There is an interlink between these concepts, in that nanosensors and nano-microbivors can be used in the detection and remediation of chemical warfare agents [146]. For example, nanosensors can be developed to detect the presence of chemical warfare agents in air or water, while nano-microbivors can be used to break down or detoxify these agents in the environment [146,147]. In this way, these technologies are important tools in ensuring national and global security.

A new wave of nanosensors is being developed to be utilized for military purposes against detection of airborne and released chemical agents that could be easily exhaled and inhaled with toxic outcomes [12,17]. Phagocytes have a cellular clearing digestive function; based on this principle, artificially designed nanoscale microbiomes are being used in studies to clean the bloodstream by digesting toxic pathogens [146]. They perform this function in a very limited time as compared to other medication options without causing any toxicity or septic shock conditions. A similar principle of action will be utilized to detect the amount of inhaled prohibited drugs such as marijuana, banned substances, and alcohol concentrations in individuals, against which the use of such substances is strictly prohibited in patients [148]. Such advanced technologies may take the place of traditional procedures, which are extensive and time-consuming diagnostic procedures.

#### 2.8.4. Nanomedicine and COVID-19

During the COVID-19 pandemic, nanomedicine has played a crucial role in developing diagnostic tools, treatment strategies, and vaccine delivery methods. The link between the coronavirus and nanoparticles based on size and function is relatively straightforward. In terms of size, both the virus particles and nanoparticles are tiny particles with a size on the nanoscale [149]. This small size allows them to interact with each other on a very tiny scale. Similarly, in terms of functional similarities, nanoparticles can be engineered or designed to have specific functions. For example, some nanoparticles can be coated with molecules that make them stick to viruses such as the coronavirus [150]. This function is essential because it allows nanoparticles to “grab onto” the virus. Thus, in the context of the coronavirus, scientists have explored how nanoparticles can be used in various ways including detection, treatment, and protective responses. Nanoparticles can be designed to bind to specific parts of the coronavirus. When they attach to the virus, they can change color or emit light, making it easier for scientists and doctors to detect the presence of the virus in a sample, such as a patient’s blood or saliva [151]. Similarly, nanoparticles can also be used to deliver medicines directly to the virus or infected cells. Think of nanoparticles as tiny delivery vehicles that can carry antiviral drugs right to the site of infection, potentially making treatments more effective [152]. In addition, regarding the protective technologies against COVID-19, Some masks and face coverings have been designed with nanoparticle coatings that can trap and neutralize viruses, including the coronavirus, when they come into contact with the mask’s surface [149,151]. Furthermore, nanoparticles have been used to create highly sensitive and specific diagnostic tests that can detect SARS-CoV-2 in patient samples [149]. Nanoparticles have also been used to develop therapeutics that can directly target the virus, as well as improve the delivery and efficacy of existing drugs [149].

In addition, nanotechnology has been used to improve the stability and efficacy of vaccines, as well as develop new delivery methods such as nasal sprays and microneedle patches [149,150]. These approaches can help increase vaccine accessibility and effectiveness, particularly in resource-limited settings. The breakthrough and rapid responses coming from nanomedicine can be ascertained by the fact that nanotechnology is also being utilized for vaccine drug manufacturing technologies against COVID-19 [151]. Since nanomedicine has already proven its benefits for disease diagnosis, treatment, and prevention, it is being employed to tackle the pandemic. Now, nano-based technology is on hand and is being considered for utilization in manufacturing antiviral technology to integrate into personalized medical equipment and to manufacture nano-based drugs [150,151]. The sole purpose is the greater safety of medical workers and to save patients suffering from the impediments of the coronavirus with more sensitive medicine and machinery.

**Figure 5:**
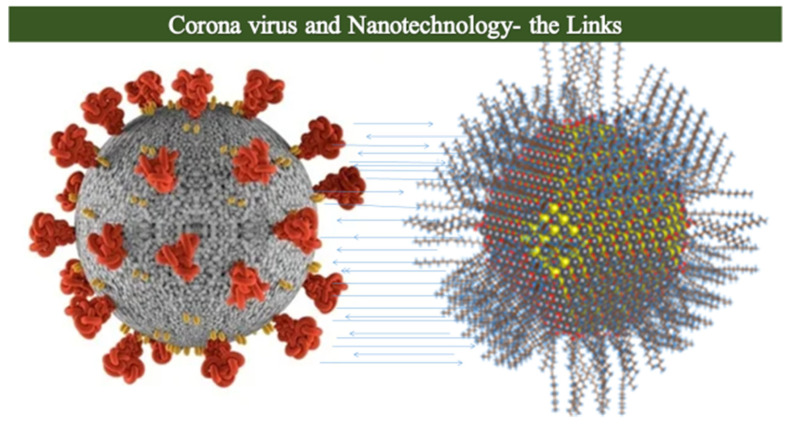
A link between coronavirus and nanoparticles based on size and function.

In this regard nanomaterials, such as quantum dots, are being introduced into biosensors for diagnostics experiments and other nanoassemblies, such as liposomes, polymeric and lipid nanoparticles, metallic nanoparticles, and micelles, which are being utilized for antiviral drug encapsulation and drug conjugation [150,151,152,153]. The great benefit would be increased pharmacological impact and more efficient drug targeting. Studies are showing that these antiviral properties of nanoparticles function by blocking the binding, entry, and replication of coronavirus in the body [154]. With this technology, the toxicity linked to normal body cells owing to nanoparticle application is the major factor of concern and thus needs to be investigated and improved for future applications [155]. Overall, nanomedicine holds great promise in the fight against COVID-19 and could potentially revolutionize the way we diagnose, treat, and prevent infectious diseases in the future. Figure 5 below shows the link between nanoparticles and coronavirus in terms of the chemistry of the structure, size, and functionality that could be used as an exemplary overview as to how nanotechnology could be majorly utilized to discover antiviral treatments in the future. Commercial applications of nanotechnology in medical field are summarized in Table 2.

### 2.9. Toxicology and Safety Analyses of Nanotechnologies

The side effects of nanotechnology are of great concern for humans, animals, and the overall environment. While the toxicity attached to these assemblies is poorly understood, the scientific community remains unsure as to what level they can extend the applications of nanotechnology, especially in medicine, which is quite a sensitive domain of healthcare [142]. In previous years, some nano-based products were introduced but later pulled back from the market owing to the reported side effects in the general public. The risk assessment of nanomedicine is thus a critical topic and needs to be assessed soon [145].

The need is to prioritize experiments for nanoparticle safety, dosing adjustment, and usage. The miracles of nanotechnology itself can be used in sensors and markers for biological, chemical, and environmental remediations [162]. Toxicity profiling of consumer products should be specifically carried out. Skin care and dental products containing different nanomaterial liposomes, cubosomes, solid lipid nanoparticles, and dendrimers must be specifically assessed, and their side effects must be determined so that more modified, effective, and harmless nanoemulsions can be introduced and utilized in the future [163].

Similarly, the issue of bioaccumulation and persistence is attached to nanotechnology. Nanomaterials have the potential to persist in the environment for extended periods and accumulate in living organisms [162,163,164]. This can lead to potentially adverse effects on both human health and ecosystems. Additionally, in healthcare settings, medical professionals who handle nanomaterials may be at risk of potential exposure through inhalation, dermal contact, or ingestion. Safe handling practices and adequate protective measures must be implemented to minimize exposure risks [165]. Moreover, the use of nanomaterials in medical applications also raises ethical considerations regarding informed consent, privacy, equity of access, and potential impacts on vulnerable populations. There is a need to address these ethical concerns to ensure the fair and responsible use of nanomaterials in healthcare [166].

To ensure the safe and sustainable use of nanomaterials in the medical field, several measures can be implemented, such as rigorous and comprehensive risk assessments, which should be conducted to evaluate the potential hazards and risks associated with specific nanomaterials before their deployment in medical applications. Similarly, adequate regulatory frameworks should be in place to ensure the safe production, handling, and utilization of nanomaterials [142,166]. This includes the evaluation of their safety, labeling requirements, and monitoring of their effects in healthcare settings. Additionally, standardized testing methods should be developed to assess the safety and efficacy of nanomaterials for medical use. This includes standardized protocols for toxicity testing, characterization, and quality control. Furthermore, strict control measures should be implemented to minimize occupational exposure to nanomaterials [165]. This includes the use of engineering controls, personal protective equipment, and employee training programs. Moreover, transparent communication about the potential risks and benefits associated with nanomaterials is essential for establishing trust among stakeholders, including healthcare professionals, patients, and the general public [165,166].

The need is to prioritize experiments for nanoparticle safety, dosing adjustment, and usage. The miracles of nanotechnology itself can be used to produce sensors and markers for biological, chemical, and environmental remediations [166]. Toxicity profiling of consumer products should be specifically carried out. Skin care and dental products containing different nonmaterial liposomes, cubosomes, solid lipid nanoparticles, and dendrimers must be specifically assessed, and their side effects must be determined so that more modified, effective, and harmless nano-emulsions can be introduced and utilized in the future [145,166]. Overall, by evaluating potential risks, implementing appropriate regulatory measures, and promoting responsible use, nanomaterials can be safely and sustainably utilized in the medical field for improved diagnostics, drug delivery, and disease treatment.

### 2.10. Future Prospects Regarding Nano-Medical Applications

Nanomaterials hold significant promise for various biomedical advancements and industrial applications. However, their unique physicochemical properties raise concerns about their potential impact on human health and the environment. In order for medical nanomaterials to enter the market, there are many obstacles to overcome, such as FDA certifications and permits, as well as safety and ethical concerns. In recent years, regulatory bodies worldwide have focused on developing appropriate frameworks to ensure the safe and responsible use of nanomaterials. Such an issue should be addressed more intensively in the coming years of nanotech research. Review papers, in this regard, should aim to provide researchers, policymakers, and industry professionals with a comprehensive understanding of the recent regulatory affairs surrounding nanomaterials. By critically examining the current state of nanomaterial regulation, this paper highlights the need for harmonization and collaboration among regulatory agencies worldwide. Regulating industrialization affairs surrounding nanomaterials in medical sciences involves several steps. It is important to note that these steps provide a general framework, but the specific details and processes may vary depending on the jurisdiction and specific requirements of each country or region. A general outline of the process is provided in a table format (Table 3) below. Steps needed to regulate the industrial affairs of nanotechnology are shown in Table 4.

## 3. Materials and Methods

A comprehensive search strategy was adopted for this systematic review to include data from diverse, recent, and the most cited sources of study.

### 3.1. Search Strategy

Data were collected via a systematic literature search through various online sources including Google Scholar, PubMed, NIH (National Library of Medicine), Web of Science, European database, Springer, and Embase databases. Since the study was focused on the applications of nanotechnology in medicine and healthcare, the major research items were “nanotechnology”, “nanobiotechnology”, “nanomedicine”, “nanotechnology and medical applications”, “nanotechnology and diagnosis”, “nanotechnology and treatment”, “nanotechnology and drug-delivery”, and “nanotechnology and healthcare and esthetics”, among other similar search terms. After a thorough analysis of titles and abstracts of publications related to applications of nanotechnology in the medical and healthcare industry, the data was selected to be part of this study. Only studies published in the English language were included in this study. Moreover, only data from 2010 onwards were included in the article.

### 3.2. Inclusion and Exclusion Criteria

Multiple types of sources were used, including data from research articles, book chapters, review articles, case reports, clinical trials, and case studies published starting beginning in 2010. Studies with incomplete citations and published before 2010 were excluded from the study.

## 4. Conclusions

The future of nanotechnology in healthcare and medicine holds immense potential for revolutionizing the way we diagnose, treat, and prevent diseases. Nanotechnology involves the manipulation of materials at such a small scale where the properties of materials significantly differ from their bulk counterparts, allowing for precise control of their physical, chemical, and biological properties. This opens up new opportunities for developing novel therapies, targeted drug delivery systems, and sensitive diagnostic tools. In addition to drug delivery, targeted delivery, improved drugs, limited dosages, and reduced systematic side effects, nanoparticles can also be used to enhance the efficacy of existing drugs by improving their solubility, stability, and bioavailability. Additionally, nanotechnology-based sensors and devices can monitor patient health in real-time, enabling early detection and personalized treatment plans. In the future, nanotechnology may even enable the development of nanorobots that can navigate through the bloodstream to target and destroy cancer cells or deliver payloads of drugs to particular tissues.

The broad spectrum of nanomedicine covered in this article may be lacking in various other aspects of nanomedicine still in the research pipeline. The vision of nanotechnology might seem heretic and abstract, similar to the in silico experimentation and computational bioinformatics field that was criticized a few years back. However, the field of nanobiotechnology is rapidly appearing as a cutting-edge technology of the 21st century, with diverse implications in science and technology. The theoretical knowledge is there, and applied research is ongoing to make it more progressive. It is predicted that soon, nanotechnology will not remain an option but rather be compulsory in the medical industry. As soon as the cost associated with technology becomes accessible, it is predicted to affect our dentistry, healthcare, and human life more profoundly than in the past. The major need is to curtail the toxicological concerns and risks that are attached to high doses and the excessive use of nanomaterials in drug and treatment regimes. This is important if scientists want to enable the successful operation of nanotechnology in medicine. Overall, the future of nanotechnology in healthcare and medicine holds great promise for improving patient outcomes and revolutionizing the way we approach disease prevention and treatment.

## Figures and Tables

**Table 1:** Barriers associated with nano-based drug delivery.

Sr. No.	Barriers Associated with Nano-Based Drug Delivery	Brief Explanation	Examples	References
1.	Size and stability	The size of nanoparticles plays a crucial role in their delivery efficiency. Very small nanoparticles may be cleared quickly from the bloodstream, while larger nanoparticles may have limited tissue penetration. Additionally, maintaining the stability of nanoparticles during storage and delivery can be challenging.	Liposomal nanoparticles although initially promising for drug delivery, have faced challenges due to their size variability and instability, leading to a limited clinical translation.	[15,16,35]
2.	Controlled release	Efficient release of the encapsulated drug or payload at target site is critical. It requires precise control over the release mechanism, kinetics, and release triggers (pH, temperature, and enzymes) to ensure optimal therapeutic effects.	Researchers working on tumor-targeted drug delivery have faced challenges in achieving the controlled release of drugs from nanoparticles thereby reducing their therapeutic efficacy.	[16,17]
3.	Targeting specificity	Nanoparticles often require functionalization with targeting ligands to enhance their specificity toward diseased cells or tissues. Achieving selective targeting while minimizing off-target effects remains a significant challenge	Gold nanoparticles functionalized with antibodies for cancer-targeted photothermal therapy have faced issues related to non-specific accumulation and targeting of healthy tissues, leading to toxicity concerns	[66,74]
4.	Biocompatibility and toxicity	Nanoparticles should be biocompatible to avoid adverse reactions and toxicity. This includes minimizing immune responses, toxicity to healthy tissues, and ensuring nanoparticles do not accumulate excessively in the body.	Carbon nanotubes showed significant toxicity concerns due to inflammation and tissue damage, limiting their clinical translation despite their potential applications.	[74]
5.	Scalability and manufacturing	The development of nanoparticles suitable for large-scale production and manufacturing can be challenging. Ensuring reproducibility and stability across batches is crucial for clinical translation.	Promising nanoparticle formulations have failed to progress to clinical trials due to scalability issues and the inability to reproduce them consistently at larger scale.	[68,74]
6.	Regulatory approval	Nanoparticles as drug delivery systems require rigorous regulatory assessment for safety, efficacy, and quality control. Meeting the extensive regulatory requirements demanded for clinical translation can be lengthy and resource-intensive.	The regulatory approval process for nanoparticles such as liposomes or polymeric nanoparticles often involves long and complex pathways, delaying their clinical translation.	[35]

**Table 2:** Examples of nanotechnological applications and their commercialization in the medical field.

Sr. No.	Examples of Nanotechnological Applications and Their Commercialized Cases in Medical Field	Brief Explanation	References
1	Drug delivery systems	Nanoparticles can be used to deliver drugs directly to targeted areas, improving their efficacy and reducing side effects. Examples include Abraxane (paclitaxel nanoparticles) and Doxil (liposomal doxorubicin).	[5,15,24,28]
2	Cancer diagnostics	Nanotechnology-based platforms can detect cancer biomarkers with high sensitivity and specificity. One example is MagArray, a magnetic nanotechnology-based biosensor for breast cancer diagnosis.	[113,114,115]
3	Tissue engineering	Nanomaterials such as nanofibers and nanocomposites can be used to construct artificial tissues and scaffolds to promote tissue regeneration and repair, as seen in the commercialized case of CardioCel for cardiovascular tissue repair.	[16,17,156]
4	Imaging agents	Nanoparticles can enhance the contrast of medical imaging techniques such as MRI, CT, and PET scans. Feridex (iron oxide nanoparticles) is an example of a commercialized MRI contrast agent.	[19,133,157,158]
5	Antibacterial coatings	Nanoscale antibacterial agents can be incorporated into medical devices such as catheters to prevent infections. The commercial product Nano-Silver Catheter is one such example.	[6,69,70,77]
6	Diagnostic nanoparticles	Quantum dots or gold nanoparticles can be engineered to detect and quantify target molecules, enabling highly sensitive medical diagnostics. ClearLight™ Diagnostics uses quantum dots for molecular imaging in tissue diagnostics.	[118,129,155]
7	Biosensors	Nanofabricated sensors can detect disease-related biomarkers and monitor conditions in real-time, such as glucose monitoring devices for diabetes management, for example, FreeStyle Libre.	[12,17,20,21,26]
8	Wound healing	Nanofiber-based dressings and coatings can accelerate wound healing by promoting	[159]
9	Chemotherapy	Nanoparticles can be loaded with therapeutic agents such as chemotherapy drugs or gene therapies, allowing targeted treatment of cancer cells. Examples include Doxil (liposomal doxorubicin) and Onivyde (nanoliposomal irinotecan).	[4,16,32,39,40,160]
10	Regenerative medicine	Nanomaterials can stimulate tissue regeneration and repair, such as the commercialized product BioCartilage for osteochondral defects.	[73,79,85]
11	Artificial organs	Nanotechnology can assist in designing and fabricating artificial organs with improved functional properties. The HeartWare™ Ventricular Assist System is a commercialized example for heart failure patients.	[85,161]
12	Early disease detection	Nanosensors can detect early-stage diseases through biomarker analysis, potentially enabling early intervention and improved outcomes. CarisomeOvarian is a nanosensor-based test for early detection of ovarian cancer.	[16,19,52,92,93]
13	Nanorobots for targeted therapy	Tiny nanorobots can be engineered to perform specific medical tasks, such as delivering drugs or unclogging.	[91,92,99]
14	Dental applications	Nanomaterials are used in dental restoration materials, such as nanocomposites	[99,100,101,102,103,104,105]
15	Drug discovery	Nanotechnology enables high-throughput screenings and drug design methods, accelerating the discovery of new therapeutic compounds. The commercialized product Nanotax uses nanotechnology for drug discovery.	[112,115,116,117,118,119,120]

**Table 3:** FDA approved and commercialized nanomedicines.

Sr. No.	Names of Products	Brief Explanation	References
1.	Patisiran (Onpattro^®^)—FDA-approved lipid nanoparticles (LNPs) are a type of delivery system used in RNA interference (RNAi) drugs. RNAi is a biological process that regulates gene expression by silencing specific genes.	Patisiran is used to treat hereditary transthyretin-mediated amyloidosis (hATTR), a rare genetic disease. It works by targeting and silencing the gene responsible for producing transthyretin, a protein that forms abnormal amyloid deposits in tissues such as the nerves and heart. LNPs are crucial in delivering a small piece of RNA (siRNA) that specifically binds to and prevents the production of the disease-causing transthyretin protein.	[164,167,168,169]
2.	Comirnaty^®^ and Spikevax^®^—both are mRNA-LNPs, based COVID-19 vaccines	Comirnaty^®^ is the brand name for the mRNA-LNP vaccine developed by Pfizer-BioNTech, while Spikevax^®^ is the mRNA-LNP vaccine produced by Moderna. These vaccines use messenger RNA (mRNA) technology to provide instructions to the body’s cells to produce a harmless piece of the spike protein found on the SARS-CoV-2 virus. The LNPs in these vaccines act as delivery vehicles to protect and transport the mRNA into the cells. The cells then use these instructions to produce the spike protein, enabling the immune system to recognize and mount a defense against the spike protein. This immune response prepares the body to defend against a subsequent infection with the actual SARS-CoV-2 virus.	[160,161,164,167,168,169]

**Table 4:** Steps needed to regulate industrialization affairs surrounding nanomaterials in the medical sciences.

Sr. No.	Steps Needed for Industrial Regulation	Brief Explanation	References
1	Risk assessment:	There is need to conduct a comprehensive risk assessment to understand the potential risks associated with the use of nanomaterials in medical applications. Evaluate the toxicity, exposure pathways, and potential environmental impacts of these materials.	[170]
2	Regulatory framework	Develop a regulatory framework specifically tailored to govern the industrialization of nanomaterials in medical sciences. This framework should consider existing regulations and guidelines but also address the unique properties and potential risks posed by nanomaterials.	[170,171,172]
3	Classification and characterization	Establish criteria for classifying and characterizing different types of nanomaterials used in medical sciences. This should include their physical and chemical properties, intended uses, and potential risks. This information will assist in determining appropriate regulations and handling requirements	[170,173]
4	Product registration and safety assessment	Introduce a registration or approval process for nanomaterials used in medical applications. Manufacturers must submit detailed information about the materials, including their synthesis methods, intended applications, potential hazards, and safety data. Conduct a thorough safety assessment based on this information before granting approvals.	[173,174]
5	Labeling and traceability	Implement labeling requirements to ensure proper identification and traceability of medical products that contain nanomaterials. Labels should provide clear information about the presence of nanomaterials, their type, concentration, and any potential risks associated with their use.	[174,175]
6	Manufacturing standards	Establish manufacturing standards and best practices specifically for nanomaterials used in medical applications. These standards should address issues such as quality control, handling, storage, transportation, and waste management, considering the unique properties of nanomaterials.	[170,173]
7	Monitoring and surveillance	Develop a surveillance system to monitor the usage, performance, and safety of nanomaterials in medical sciences. Regularly review and update regulations based on emerging scientific evidence, advancements in technology, and any new risks identified.	[174]
8	Collaboration and international harmonization	Foster collaboration and information-sharing initiatives with other regulatory bodies and international organizations to harmonize standards and regulations for nanomaterials used in medical sciences. This will help avoid duplication of efforts, facilitate global trade, and ensure a consistent level of safety worldwide.	[175]
9	Public engagement and communication	Engage the public, stakeholders, and healthcare professionals in the regulatory process. Conduct public consultations, disseminate information about regulations, and address concerns raised by various stakeholders. Effective communication will help build trust and ensure transparency in the regulation of nanomaterials in medical sciences.	[175]

## Data Availability

Not applicable.
